# Air pollution and health in Sri Lanka: a review of epidemiologic studies

**DOI:** 10.1186/1471-2458-10-300

**Published:** 2010-06-02

**Authors:** Yatagama Lokuge S Nandasena, Ananda R Wickremasinghe, Nalini Sathiakumar

**Affiliations:** 1 Evaluation and Research Unit, National Institute of Health Sciences, Ministry of Health, Kalutara, Sri Lanka; 2 Department of Public Health, Faculty of Medicine, University of Kelaniya, Kelaniya, Sri Lanka; 3 Department of Epidemiology, School of Public Health, University of Alabama at Birmingham, Birmingham, USA

## Abstract

**Background:**

Air pollution is increasingly documented as a threat to public health in most developing countries. Evaluation of current air quality levels, regulatory standards and scientific literature on outdoor and indoor air pollution, and health effects are important to identify the burden, develop and implement interventions and to fill knowledge gaps in Sri Lanka.

**Methods:**

PUBMED and Medline databases, local journals and conference proceedings were searched for epidemiologic studies pertaining to air pollution and health effects in Sri Lanka. All the studies pertaining to air pollution and health effects were considered.

**Results:**

Sixteen studies investigated the association between exposure to ambient or indoor air pollution (IAP) and various health outcomes ranging from respiratory symptoms, low birth weight and lung cancers. Of the sixteen, three used a case control design. Half of the studies collected exposure data only through questionnaires. There were positive associations between air pollution and adverse health effects in all studies. Methodological limitations in most of the studies resulted in poor quantification of risk estimates.

**Conclusion:**

A limited number of epidemiological studies in Sri Lanka have investigated the health effects of air pollution. Based on findings of studies and reported air quality levels, air pollution may be considered a neglected public health problem in Sri Lanka.

## Background

Air pollution, both indoors and outdoors, is a major environmental health problem affecting people in both developed and developing countries. Although air pollutants are many, the most important are particle pollution (often referred to as particulate matter (PM)), ground-level ozone (O_3_), carbon monoxide (CO), sulfur oxides (SO_x_), nitrogen oxides (NO_x_), and lead (Pb) which are found in the ambient air (also known as "criteria pollutants"); PM, CO, SO_x_, NO_x_, environmental tobacco smoke (ETS), formaldehyde and polycyclic organic matter are found indoors [1,2].

Exposure to air pollutants leads to a variety of health effects depending on the type of pollutant, amount of the pollutant exposed to, duration and frequency of exposure, and associated toxicity of the specific pollutant. These exposures are associated with a broad range of acute and chronic health effects varying from sub-clinical effects to premature mortality [3]. Although air pollutants are categorized in a number of different ways, most air pollutants generally do not occur in isolation, but in complex mixtures that create the potential for synergistic effects among them [4]. The composition of air pollutants and their associated toxicity vary in different settings. Age, cultural practices, life style and socio-economic status may influence the exposure to air pollutants [4]. As the individual sensitivity to pollutants increases, the severity of the response will increase for a given pollutant. Therefore, the effects of air pollutants and the severity of health outcomes in a given population depend on the population sensitivities [3]. Hence, the health impact of air pollution on a given community cannot be directly generalized from results of studies in other settings.

Several population groups are more vulnerable to the effects of air pollutants; those who are innately susceptible more than others, those who become susceptible as a result of environmental, social and personal behaviours, and those who are simply exposed to unusually large amounts of air pollutants [5]. Groups that are more sensitive to air pollutants include unborn and young children, elderly people, and those with a history of cardio-respiratory diseases [5]. Hence, the demographic profile of a given population is also important.

Although air pollution is recognized as an emerging public health problem in developing countries, most of these countries do not have adequate data to evaluate the actual magnitude of the problem. The main reason may be that air pollution co-exists with other important public health problems, such as communicable diseases, vector-borne diseases, malnutrition and poor sanitation, which are given higher priority in circumstances where economic resources are limited. This has delayed the actions needed to adequately assess, evaluate and control air pollution [4]. The Sri Lankan situation is no different to other developing countries.

Our primary interests were to explore the air quality levels and review the literature regarding health effects due to air pollution in Sri Lanka. In the first part of this review, we discuss air quality, control measures implemented, and air quality regulations in Sri Lanka separately for ambient and indoor air. Secondly, we review the studies pertaining to health effects due to air pollution in Sri Lanka.

## Ambient air pollution

Ambient air pollution, especially in urban environments, arises from a spectrum of different sources, which are broadly classified as stationary, mobile, and area emission sources. The main source of ambient air pollution in Sri Lanka is vehicular emissions, which contributes to over 60% of total emissions in Colombo [6].

Measures have been taken to reduce outdoor air pollution due to vehicular emissions. The National Policy on Urban Air Quality Management was adopted in 2000. The phasing out of leaded gasoline in June 2002, introduction of low sulphur diesel in January 2003, banning the importation of Two Stroke Three-wheelers in 2008, and initiation of vehicular emission testing programme in year 2008 are some key steps that have been taken to control urban outdoor air pollution in Sri Lanka. The permissible ambient air quality standards for selected air pollutants were for the first time enacted under the National Environmental (Ambient Air Quality) Regulations of 1994 [7]. With the publication of WHO air quality guidelines in 2005[8], air quality standards for Sri Lanka, including standards for PM_10 _and PM_2.5, _were amended and gazetted in August 2008[9] (Table 1).

**Table 1:** Sri Lankan air quality standards

Air pollutant	Average time	Sri Lankan standard (μg/m^3^)
Carbon monoxide	8 hr	10000
	
	1 hr	30000
	
	Any time	58000

Nitrogen dioxide	24 hr	100
	
	8 hr	150
	
	1 hr	250

Sulfur dioxide	24 hr	80
	
	8 hr	120
	
	1 hr	200

Ozone	1 hr	200

Lead	Annual	0. 5
	
	24 hr	2

SPM	Annual	100
	
	24 hr	300
	
	8 hr	350
	
	3 hr	450
	
	1 hr	500

PM_2.5_	24 hr	50
	
	Annual	25

PM_10_	24 hr	100
	
	Annual	50

**Figure 1:**
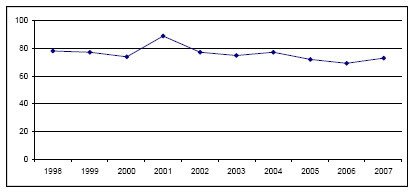
**Annual averages of PM-10 at Colombo Fort ambient air quality monitoring Station (1998-2007)**. Source: Central Environmental Authority (Year 2007).

**Figure 2:**
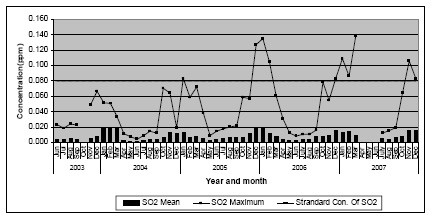
**Monthly mean sulfur dioxide concentrations at Colombo Fort (June 2003 - December 2007)**. Source: Central Environmental Authority (2007).

**Figure 3:**
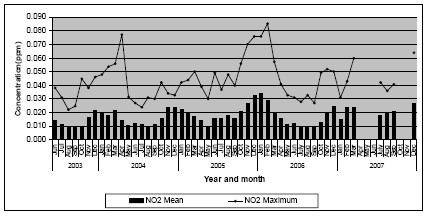
**Monthly mean of one-hour averages of nitrogen dioxide concentrations at Colombo Fort (June 2003 - December 2007)**. Source: Central Environmental Authority (Year 2007).

Despite many discussions to expand air quality monitoring in Sri Lanka, there has been only one station located at Colombo Fort, since 1997, to monitor ambient air quality on a continuous basis. Based on data from this station, the average annual ambient PM_10 _levels in Colombo over the years have remained relatively stable ranging from 72 to 82 μg/m^3 ^(Figure 1). The World Health Organization (WHO) recommends that the average annual ambient PM_10 _level be <20 μg/m^3^[8]. Comparison of coarse particle mass concentrations of the Asian region under the Regional Co-operation Agreement (RCA) shows that the three-year average values reported from Colombo Fort (an urban location) was 73.37 μg/m^3 ^and in a residential area was 58.82 μg/m^3 ^from 2002 to 2005. Special 24-hour measurements for the purpose of the inter-country comparison were carried out at the Colombo Fort monitoring station on a weekly basis using the Gent stacked filter unit particle samplers. The values reported from other regional locations (used the same methodology and instruments) were: Bangladesh urban location - 45.76 μg/m^3^; India-Trombay - 37.34 μg/m^3^; India-Vashi - 82.83 μg/m^3^; Pakistan - 67.45 μg/m^3^, Thailand, urban - 38.67 μg/m^3^, Thailand, suburban - 25.77 μg/m^3 ^and Vietnam - 50.29 μg/m^3 ^[10]. Another comparison of several cities around the world reported that the PM_10 _and SO_2 _levels in Colombo (based on the Colombo Fort monitoring station) is unhealthier than cities such as Hong Kong, Singapore, Bangkok Taipei and Tokyo, and the PM_10 _levels in Colombo are similar to that of Ho Chi Minh, Jakarta and Mumbai [11]. Hourly averages of SO_2 _have exceeded the Sri Lankan standards on 177 occasions from May 2003 to December 2006. The annual mean levels of SO_2 _have not exceeded the USEPA limit of 80 μg/m^3^, but the levels have shown an increasing trend in the last few years (Figure 2). NO_2 _levels in the Colombo city over the past few years have shown an increasing trend although the levels did not exceed the Sri Lankan standards (Figure 3). High levels of air pollutants were recorded during the North East monsoon period which lasts from mid November to January over the years [12]. CO and O_3 _levels were relatively low in Colombo as compared to other air pollutants [13]. Many criticisms have been expressed with reference to the Colombo Fort monitoring station underestimating air quality in the Colombo city area. Some of the criticisms include the location of the monitoring station being not more than 800 m from the sea, which causes the shedding off of pollutants due to wind, shifting of high traffic towards the interior of the country due to prevailing high security zone in its vicinity, and newly established factories and power plants being situated several kilometres away from the monitoring station.

The National Building Research Organization of Sri Lanka established a passive air quality monitoring network in 2001 covering 15 locations in the Colombo city to monitor SO_2 _and NO_2_[14]. Mallikarachchi et al [15], and Manawadu and Wijesekara [16] showed that the air quality of Colombo city was significantly associated with land transport density, population and building density by modelling air quality in the Colombo city, using data from the passive sampling air quality monitoring network.

## Indoor air pollution

Indoor exposures are a result of complex interactions between the structure, building systems, indoor source strength, removal and deposition rate within the structure, indoor mixing and chemical reactions, furnishings, the outdoor environment, and the practices and the behaviours of the inhabitants [17]. High pollutant concentrations can result inside a closed indoor environment even with relatively modest emissions. When the windows are open and the wind speed is moderate or high, the indoor concentrations of air pollutants may be the same as that outdoors [18]. Although Sri Lanka being a tropical country is expected to have good indoor ventilation, limited data suggest that indoor air is more polluted than outdoor air [19,20].

Cooking fuel is the main source of IAP in households [21] while other sources include tobacco smoke and smoke from other outdoor sources [1]. The types of fuel typically increase in cleanliness, convenience, efficacy and cost as people move up the "energy ladder" [1]. According to the Demographic and Health Surveys of Sri Lanka of 2000 and 2007, firewood is the principal type of cooking fuel in 78.3% and 78.5% of the households in Sri Lanka, respectively [22]. It is unlikely that a higher proportion of Sri Lankans will shift to cleaner fuels in the near future [23]. Most of the traditional local stoves using firewood have incomplete combustion resulting in high pollutant emissions. This coupled with poor ventilation can produce very high levels of indoor pollution [24]. A study assessing exposure in kitchens using firewood with traditional stoves reported average PM_2.5 _concentrations exceeding 1200 μg/m^3 ^[24]. A reduction of IAP was shown by the use of improved wood stoves in various settings in Sri Lanka [24] although the use of these stoves at the community level is almost negligible. Women and young children who usually stay around their mothers while cooking may be the most vulnerable to IAP due to cooking fuel.

A cross sectional study conducted to measure SO_2 _and NO_2 _levels indoors and outdoors of 30 low-income households at five different locations in Colombo reported that indoor pollutant levels were higher than those outdoors in all households. In that study, parents maintained a daily activity diary for their 5 to 8 year-old children which showed that these children spent an average 41 hours (out of 48 hours) inside their houses during weekends. The health status of children was not assessed in this study [25]. Regular use of mosquito coils by 12% of households may be another source of IAP [22] especially in poorly ventilated houses; the health impact of this exposure has not been investigated in Sri Lanka.

The National Authority on Tobacco and Alcohol (NATA) Act [26] banned smoking in health-care institutions, educational institutions, government facilities, universities, indoor offices, and other indoor workplaces. The Global Youth Tobacco Survey (GYTS) reported that there is no marked reduction in second-hand smoke in public places following the implementation of the NATA Act [27]. The GYTS further reported that there is a reduction in the prevalence of parental smoking from 1999 (50.8%, 95%CI = 47.8%-53.8%) to 2007 (29.9%, 95% CI = 25.6%-34.5%). With almost one third of parents currently smoking, second-hand smoke in households may still be high and need to be explored quantitatively.

## Methods

### Search strategy

We searched literature from PUBMED and MEDLINE databases to identify all relevant publications prior to June 2009. The following search terms were used: "ambient air pollution AND Sri Lanka", "indoor air pollution AND Sri Lanka", "air pollution AND Sri Lanka", "epidemiologic studies AND Sri Lanka", "health effects AND Sri Lanka."

We manually identified additional studies published in local journals and included conference proceedings which were inaccessible via the electronic databases. Reference lists of original studies were checked for related studies (cross reference). Authors of selected studies were contacted to identify additional literature.

### Study selection

We included all studies relating to health effects due to air pollution in Sri Lanka irrespective of the study design. Studies that actually measured air quality levels and studies using proxy variables to predict air quality, indoors, outdoors or both, were included. We excluded studies which did not have health outcomes, and studies presented as conference proceedings which were not supplemented with a full report. We considered only the studies published in English as there are no scientific journals or any scientific literature available in the local languages. Preliminary results of ongoing studies were not considered.

### Data extraction

Two investigators reviewed each paper independently. Discrepancies were discussed and agreement was achieved with consensus. Papers were categorised as indoor or outdoor based on the methodology. Studies were further categorised according to the study design. The following information from each study was abstracted: study location, exposure assessment, health outcomes, other risk factors evaluated, study results and limitations.

## Results and Discussion

### Study Characteristics

**Figure 4:**
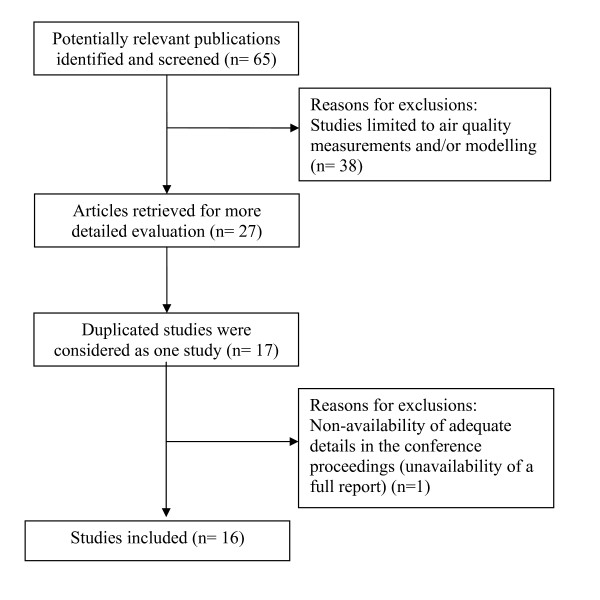
**Flow diagram of selection of studies**.

We retrieved 65 publications and conference proceedings of potential interest, of which, 38 were excluded initially as they did not have a health outcome resulting from air pollution. Duplications of studies as publications and conference proceedings were taken as a single study. We finally identified 17 original studies. One study was excluded as we were unable to find the full report of the abstract presented in the conference proceeding (Figure 4). Studies included are summarized in Tables 2 &3. Ten and six studies focussed on outdoor air pollution (Table 2) and indoor air pollution (Table 3), respectively. Nine studies had used a cross-sectional study design. Most of the health outcome studies show positive associations between air pollution and various adverse health effects, which correspond to results from studies conducted in other countries [1,5]. However, some studies have methodological limitations due to lack of objective measurement of personal exposures and indoor air quality leading to potential misclassification. Apart from age and sex, most studies do not adjust for potential confounding factors such as sources of air pollution other than those described in the study. Furthermore, possible factors which may influence indoor air pollution levels such as ventilation of houses, location of the kitchen, stove types and availability of exhaust mechanisms to expel the generated smoke from biomass fuel combustion, smoking inside households and mosquito coil use were poorly quantified (Figure 4).

**Table 2:** Studies assessing the health effects of ambient air pollution in Sri Lanka

Reference, study location and data collection period	Study design, subject characteristics and sample size	Exposure air pollutants	Health outcomes	Results	Adjustment for confounding factors	Limitations
**Cross sectional studies**						
Premaratna R et al. [29], Gampaha district. 1997	Children, 1-12 years (n = 154); adults (n = 304)	Not measured	Respiratory symptoms & peak flow rate	Higher rate of respiratory symptoms and reduction in expiratory flow rate reported in the industrialized area.	No	No measurement of exposure
						
Senanayake MP et al.[37], Colombo. 1998 and 2003	Children, 1-15 years (1998, n = 50; 2003, n = 39)	Blood lead levels	Blood lead levels	6% of children had blood lead levels >10 μg/dl when leaded petrol was used (1998); none had levels > 10 μg/dl, one year after unleaded petrol was introduced.	No	Small sample size. Comparison of different birth cohorts.
Amarasinghe J.N.P.et al.[38], Colombo. 2002	Policemen, traffic (n = 64); non-traffic (n = 64)	Blood lead levels	Potential symptoms and signs resulting from high blood lead levels	Abdominal discomfort, tremor and hypertension higher in traffic policemen as compared to non- traffic policemen	No	Control group may also have had a high exposure during the busy hours leading to misclassification
Mistry R. et al.[32], Galle and Chandigarh. 2004	Children, 13-14 years (Galle, n = 1162; Chandigarh, n = 575)	No specific types are measured	Wheezing	Occurrences of wheezing was higher in Galle as compared to Chandigarh	No	No measurement of exposure
Nandasena YLS et al.[30], Colombo and Ampara districts. 2005	Children, 9 - 15 years (n = 482)	SO_2_ NO_2_ PM_10_	Respiratory symptoms	Respiratory symptoms were higher in Colombo as compared to the rural area. Associations were overridden by household risk factors.	Adjusted for cooking fuel type and mosquito coil use	Only respiratory symptoms are considered
Perera GBS et al.[35], Colombo and Ampara districts. 2005	Adults (n = 587)	SO_2_ NO_2_ PM_10_	Respiratory symptoms	Occurrence of respiratory symptoms were higher in Colombo as compared to the rural area	Adjusted for cooking fuel type and mosquito coil use	Only respiratory symptoms are considered.
Elangasinghe MA et al.[31], Kandy. 2006	12-16 year school children (n = 760)	PM_10_	Respiratory health	32% of children of village school had a health indicator of 1 (a measure of perfect respiratory health) while only 8% from the city school had an index of 1.	No	Health indicator constructed by authors but not validated.
**Other Study Designs**						
Senanayake, MP et al.[33], Lady Ridgeway Children's Hospital, Colombo. 1998-1999	Ecological study, children under 12 years (n =41032)	NO_2_, SO_2_	Emergency reporting for nebulization	Episodes of nebulization positively correlated with most polluted days (p < 0.05)	No	No measurement of exposure of the children; pollutant data from single monitor in the city
Sirithunga TLJC et al.[28], Kandy district. 2004.	Follow-up study children 7-12 years (n = 1033)	SO_2_ NO_2_ Ozone	Respiratory symptoms	Occurrences of respiratory symptoms were higher in the Kandy city area as compared to the rural area.	Yes	Only outdoor passive samplers used; indoor air quality predicted with proxy variables
Thishan Dharshana KG and Coowanitwong N[34], LRH hospital, Colombo. 2008	Ecological study	PM_10_	Respiratory diseases	Diseases categories included bronchitis, emphysema and other chronic obstructive pulmonary diseases; positive correlation (r = 0.717; p = 0.01).	No	Colombo Fort monitoring station may not be representative of the whole study area.

**Table 3:** Studies assessing the health effects of indoor air pollution in Sri Lanka

Reference, Study Location and data collection period	Study Design Subject Characteristics and sample size	Exposure air pollutants	Health outcomes	Results	Adjustment for confounding factors	Limitations
**Cross sectional studies**						
Karunasekara KAW et al.[40], Gampaha district. 1998	Children, 5-11 years, asthmatics (n = 441); non asthmatics (n = 1510)	No specific types are measured	Asthma	Prevalence of asthma was significantly higher in the presence of firewood smoke	Yes	No measurement of exposure
Lankatilake KN et al.[20], Kotte Medical Officer of Health area. 1999	Households = 397 children = 604 women = 130	Respirable dust	Respiratory symptoms	Respiratory symptoms were significantly higher in houses using firewood	Yes	Only respirable dust levels were measured
Pathirane S M et al.[43]Kegalle and Kalutara districts. 2004	New borns (n = 369)	No specific types	Low birth weight	Low birth weight was associated with fuel type and kitchen characteristics	No	No measurement of exposure
**Case-control Studies**						
Karunasekara KAW et al.[39], Colombo North Teaching Hospital 1996-1997	Children 1-10 years, age matched cases and controls (n = 300)	No specific type	Asthma	Dust at home was a significant risk factor for asthma	Yes	No measurement of exposure
Perera MAKK P et al.[42], National Cancer Hospital 2004	Lung cancer patients (n = 128) and controls (n = 128)	No specific type	Lung cancer	No significant association with biomass exposure	No	No measurement of exposure
Ranasinghe MH et al.[41], National Eye Hospital, Colombo 2004	Patients with cataracts (n = 197) and controls (n = 190)	No specific type	Cataract	Cataracts significantly associated with biomass exposure	No	No measurement of exposure

### Studies on Ambient Air Pollution

We identified 10 studies (Table 2) that examined the effect of ambient air pollution exposure on various respiratory health outcomes. Of the 10, 7 studies adopted a cross-sectional design. In all studies, questionnaires and/or passive/active samplers were used to ascertain exposure. None of the studies used personal monitoring of exposure to ambient air. Other methodological limitations include small study sizes, potential misclassification of exposure, recall biases and inadequate adjustment for potential confounders.

#### Respiratory health

Sirithunga et al.[28] measured outdoor air pollutant levels in home environments of school children in an urban and a rural area in the Kandy district and assessed the respiratory symptoms over a one-year period on a daily basis using health diaries. Outdoor measurements representing a large area were obtained with passive samplers and indoor exposure relied on proxy parameters. The authors minimized recall bias by collecting information on the health conditions on a daily basis. The occurrence of cough, nasal discharge and throat irritation were 1.12 (95%CI = 1.05-1.19), 1.17 (95%CI = 1.09-1.24) and 1.48 (95%CI = 1.31-1.67) times higher among children in the Kandy urban area, respectively, as compared to those of the rural area. Lung functions were also measured but there were no significant differences in the measurements between urban and rural subjects. The major limitation was the potential for exposure misclassification.

Premarathna et al. [29] studied the effects of air pollution on health of residents in an industrial area in Sri Lanka using a cross-sectional design and reported that the adult population living in the industrial area was 2.1 times more likely to have unexplained episodic cough (95% CI = 1.13-7.09) and a significant reduction in expiratory flow as compared to the adult population living in a non-industrial area. The younger population (≤ 12 years) living in the industrial area were 2.8 (95% CI = 1.10-7.10) times more likely to have rhinitis as compared to the younger population living in the non-industrial area. These conclusions were based on the assumption that the industrialized area is more polluted compared to the control area. No air quality measurements were made to confirm this assumption.

School children are another group exposed to high levels of pollutants exaggerated by overcrowding of major schools in cities, especially in Colombo. The prevalence of respiratory symptoms among school children attending a school in Colombo situated close to a busy main road was significantly higher than that of children attending a school situated in a rural area (cough - OR = 1.33, 95% CI = 1.12-2.48; presence of phlegm - OR: 1.66, 95% CI = 1.12-2.46; wheezing - OR: 1.29, 95% CI = 0.80- 2.10). Children's exposure in the home environment was considered only by proxy parameters such as cooking fuel type and no adjustments were made for confounding factors. Air pollutant (NO_2_, SO_2, _and TSP) levels were significantly higher in the premises of the urban school as compared to the rural school [30]. The respiratory health of school children (12 to 16 years), residing in and around Kandy city (n = 770), was studied by measuring PM_10 _levels in a cross-sectional survey[31]. PM_10 _levels "outside-the-city school" were lower (3-hour average, 84 mg/m^3^) as compared to the "city school" (121 mg/m^3^). An arbitrary health indicator of 1, a measure of perfect health, was found among 32% of children of the "outside-the-city school" as compared to only 8% of children of the "city school". Misclassification of exposure is a potential limitation of the study as the total time spent at the school by a secondary school student is less than 15% for a one-year life span and the exposure levels in other living environments of the children were not considered. Further, considerable proportions of children in urban schools travel daily from rural locations with low outdoor exposure. Children from rural households may be exposed to more IAP due to more biomass fuel use in these areas.

Mistry R. et al. [32] compared the prevalence of asthma among 13 to 14 year-old children in Galle (n = 1162) (described by the authors as an unplanned, busy city with predominant use of kerosene or firewood for cooking in Sri Lanka) with Chandigarh (n = 575) (a city defined by the authors as a planned, clean and using smoke-free household fuel in India). Children in Galle were at an increased risk for life-time wheezing (Galle-28.7%, Chandigarh-12.5%; the prevalence odds ratios (POR) = 2.3, 95% CI = 1.8-2.9) and for wheezing in the previous year (Galle-21.9%, Chandigarh-10.4%, POR = 2.1, 95% CI = 1.6-2.7) as compared to children from Chandigarh.

An ecologic study examined the air pollutant levels measured at the Colombo Fort monitoring station and rates of hospital attendance for wheezing needing emergency treatment at the Lady Ridgeway Hospital for Children in Colombo. About 30,932 children required nebulizer therapy in the emergency treatment unit (median daily attendance, n = 85) during the 12-month period beginning in July 1998. The highest number of episodes of nebulization occurred on the most polluted day (with respect to SO_2 _and NO_2_) and the lowest number of nebulizations occurred on the least polluted day in a given week, in a significant number of weeks throughout the year (binomial test, p = 0.05) [33]. However, the actual exposure of all the patients admitted to this hospital probably did not represent the air quality of Colombo Fort monitoring station as the hospital is a referral centre for the whole country. Exposure data was limited to only a few pollutants.

Based on data of the Colombo Fort monitoring station, episodes of bronchitis, emphysema and other chronic obstructive pulmonary diseases had a strong association with PM_10 _levels (correlation coefficient = 0.717; p = 0.01). Nearly 20% of asthma patients who visited the Lady Ridgeway Hospital for Children in Colombo in 2005 could be attributed to exposure to PM_10 _in Colombo based on the health impact assessment software developed by WHO [34]. Although the location of the Colombo Fort monitoring station may not represent the entire area of study population, this study provides evidence for implementation of early mitigation strategies.

Air pollutants were measured using passive samplers in Mount Lavinia, a metropolitan area bordering Colombo, to measure the 24-hour average levels among city dwellers engaged in different occupations[35]. Bus drivers were exposed to more NO_2 _(57.36 μg/m^3^) and SO_2 _(82.70 μg/m^3^) as compared to trishaw drivers (NO_2 _- 50.18 μg/m^3^; SO_2 _- 78.36 μg/m^3^), shop keepers (NO_2 _- 54.91 μg/m^3^; SO_2 _- 63.29 μg/m^3^) and outdoor vendors (NO_2_- 37.66 μg/m^3^; SO_2 _- 35.25 μg/m^3^). Respiratory conditions were assessed in each participant using a questionnaire. The highest prevalence of respiratory symptoms was reported among bus drivers.

#### Blood lead levels

Following the introduction of unleaded gasoline, atmospheric lead levels reduced by 81.5%, 82% and 84% in three locations in Colombo [36]. Senanayake et al. [37] measured blood lead levels of a sample of children living near a traffic congested junction in Colombo in 1998 and then one year after the introduction of unleaded gasoline. In 1998, 6% of children had blood lead levels above 10 μg/dL; in 2003, not a single child had a blood lead level >10 μg/dl (range 1.67 μg/dl to 9.7 μg/dl).

Amarasinghe et al. [38] measured blood lead levels of traffic policemen (n = 64) and non traffic policemen (n = 64) based in Colombo. The mean blood lead levels in traffic and non-traffic policemen were 7.47 μg/dl (SD = 2.89) and 7.06 μg/dl (SD = 2.93), respectively. Abdominal discomfort, tremor and hypertension were higher in traffic policemen as compared to non-traffic policemen, although the differences were not significant. There may be exposure misclassification as non-traffic policemen are duty bound to control traffic during busy hours.

### Studies on Indoor Air Pollution

We reviewed 6 studies that have evaluated IAP and health outcomes in Sri Lanka. Of the 6 studies, 3 were case-control studies and 3 were cross-sectional studies. Five of the 6 studies used proxy measurements for IAP and no quantitative measurements were undertaken. There was no adjustment done for potential confounding factors.

#### Respiratory Health

A hospital-based case-control study [39] of 300 subjects (age-matched) found that the presence of dust at home was a significant risk factor (adjusted OR = 1.3, 95% CI = 1.02 - 2.1) for asthma. There may have been potential exposure misclassification and recall bias with cases over-reporting a "dusty environment".

A cross-sectional study conducted in a Colombo suburb reported that the use of fire wood for cooking was a significant risk factor for respiratory symptoms (OR = 1.61; 95% CI = 1.3 - 2.53) [20]. Average TSP levels in houses using firewood were 0.606 mg/m^3 ^and in houses not using firewood were 0.245 mg/m^3^. The respirable dust concentration exceeded the WHO standards in 84% of households using firewood and 54% of the households not using firewood [20].

Another school-based cross-sectional study of asthmatics (n = 441) and non- asthmatics (n = 1510) in the Gampaha district reported that the presence of firewood smoke in the bed room while cooking (OR 1.4, 95% CI 1.1-1.9), use of mosquito coils (OR 1.5, 95% CI 1.2 - 1.9), and a dusty environment (OR 1.8, 95% CI 1.4 - 2.3) significantly increased the risk of asthma. No air pollutant measurements were done. The environmental exposure assessment was based on a questionnaire and no adjustment for confounding factors was done [40].

#### Cataract

Ranasinghe et al. [41] reported an association (p < 0.05) between biomass exposure and cataract by comparing cataract patients (cases, n = 197) with patients admitted to the National Eye Hospital, Colombo, for other eye problems (controls, n = 190). The biomass exposure assessment was solely based on a questionnaire.

#### Lung Cancer

Perera et al. [42] investigated the association between exposure to biomass smoke and lung cancer. The majority of the population (more than 50%) was between the ages of 41-50 years and male; the majority of the cases were directly exposed to tobacco smoke (48%). There was an association between smoking and lung cancer (p = 0.04) but not with biomass fuels use (p = 0.10). Exposure was assessed using a questionnaire.

#### Low Birth Weight

A cross-sectional study [43] reported that the availability of a separate kitchen (OR = 2.7, 95% CI = 1.6-4.7), using less clean cooking fuel (OR = 3.9, 95% CI =1.8 - 8.5) and not having adequate ventilation in the cooking area (OR = 2.7, 95% CI =1.3 - 5.3) were significant predictors of low birth weight in the Kegalle and Kalutara districts. Air quality measurements were not reported in this study.

### Confounders

Several studies[28,30,35,39,40] adjusted for common confounders such as second-hand smoke exposure, cooking fuel type, mosquito coil use etc., proxy measures for indoor air quality. The air quality levels in studies that measured air quality [20,30,34,35] were similar to those reported in other countries.

### Strengths and Weaknesses

We did not use strict inclusion criteria as our primary objective was to explore most of the studies done in Sri Lanka relevant to the subject. We included studies that used only proxy variables as the number of studies that measured actual air quality was limited. We had no option but to include as many studies as possible due to the limited number.

### Future Perspectives

The broad range of likely health impacts of air pollution in Sri Lanka means that a variety of methods and strategies should be applied for mitigating adverse health effects and minimizing exposures. Generating baseline data related to indoor and outdoor air pollutants and human health will form the platform to address this problem at national, community and individual level. It will be the basis for advocacy, formulating mitigation strategies and minimizing exposure.

The lack of a proper air quality monitoring system to track human exposure is a major limitation. This has to be addressed to determine the impact of programmes and to identify the future directions. The availability of stringent standards by itself is of no use if the air quality that citizens are exposed to is unknown to identify areas for intervention using an evidence based approach. Therefore, establishing a modern ambient air quality monitoring network, at least covering the main busy cities in the country, is an early need.

Although several activities have been implemented to reduce outdoor air pollution, there are no specific interventions implemented at national level to reduce IAP or to minimize the exposure of vulnerable groups to indoor air pollutants. There is a lack of reliable indoor air quality data and determinants of indoor air quality in Sri Lanka, a priority that needs to be addressed when estimating the burden of disease associated with IAP. Enactment of new laws and enforcing existing laws will require reliable baseline data on indoor and outdoor air quality and health impact. Practices and other determinants that increase human exposure to air pollutants need to be identified in local communities. Robust research studies should be designed to generate individual exposure data, identify and evaluate determinants associated with air pollution exposures and to quantify the public health effects of such exposures in Sri Lanka. Public health impact of outdoor air pollution control activities should be assessed to monitor and modify such mitigation activities.

Modifying existing regulatory practices based on findings of robust research studies, strict adherence to regulations at community and household level and identifying new mitigation strategies can play a key role in minimizing the impact of air pollution on health.

## Conclusions

Despite inconsistencies, findings from Sri Lankan studies generally suggest that air pollution, both ambient and indoor, is a major public health problem in Sri Lanka. The results of these studies could be used as a base to design larger epidemiologic investigations to address the problems of measurement error, reduce uncertainties in risk estimates and identify the determinants of exposures.

## Competing interests

The authors declare that they have no competing interests.

## Authors' contributions

YLNS, ARW and NS designed the study. YLSN completed the initial literature survey. YLSN and ARW summarized all eligible papers, synthesized the findings and drafted the manuscript. NS helped in drafting the final manuscript. YLSN is the guarantor of the work. All authors read and approved the final manuscript.

## Pre-publication history

The pre-publication history for this paper can be accessed here:

http://www.biomedcentral.com/1471-2458/10/300/prepub
